# Attachment and Biofilm Formation of Eight Different *Salmonella* Serotypes on Three Food-Contact Surfaces at Different Temperatures

**DOI:** 10.3390/microorganisms13071446

**Published:** 2025-06-21

**Authors:** Katrina L. Counihan, Shannon Tilman, Joseph Uknalis, Sudarsan Mukhopadhyay, Brendan A. Niemira, Daniela Bermudez-Aguirre

**Affiliations:** 1 Characterization and Interventions for Foodborne Pathogens Research Unit, United States Department of Agriculture, Agricultural Research Service, Eastern Regional Research Center, 600 East Mermaid Lane, Wyndmoor, PA 19038, USA; katrina.counihan@usda.gov (K.L.C.); shannon.tilman@usda.gov (S.T.); sudarsan.mukhopadhyay@usda.gov (S.M.); brendan.niemira@usda.gov (B.A.N.); 2 Microbial and Chemical Food Safety Research Unit, United States Department of Agriculture, Agricultural Research Service, Eastern Regional Research Center, 600 East Mermaid Lane, Wyndmoor, PA 19038, USA; joseph.uknalis@usda.gov

**Keywords:** foodborne pathogens, interventions, sanitizing, food industry, cell attachment

## Abstract

*Salmonella* spp. represent a food safety risk in the production chain because of their potential for biofilm development. This study examined the biofilm formation of eight *Salmonella* serotypes from diverse foodborne outbreaks on three food-contact surfaces, stainless steel, silicone, and nylon, at 10 °C and 37 °C. The effect of temperature was observed in slower biofilm formation at 10 °C with about 5-log (cfu/cm^2^) after 24 h, contrasting with 7-log (cfu/cm^2^) at 37 °C. The material also influenced biofilm formation, with the strongest biofilms on stainless steel at 10 °C and silicone at 37 °C. The serotypes producing the strongest biofilms were *S.* Enteritidis, *S.* Saint Paul, and *S.* Montevideo. The weakest serotypes were *S.* Senftenberg, *S.* Anatum, and the avirulent *S.* Typhimurium. The production of extra-polymeric substances was evident with *S.* Enteritidis. The biofilm index showed the highest value for low temperature, nylon, and silicone, and for *S.* Montevideo, *S.* Enteritidis, and *S.* Saint Paul. The whole-genome sequencing of each serovar suggested that single nucleotide polymorphisms in the curli (*csg*) genes may have contributed to the strong biofilm-forming ability of *S.* Montevideo and *S.* Saint Paul and the weaker ability of *S.* Senftenberg. These results can help with the correct development of sanitizing interventions based on the *Salmonella* strain of concern.

## 1. Introduction

*Salmonella enterica* is one of the top foodborne pathogens in the world, linked to several food products generating diseases, hospitalizations, deaths, and economic losses. The top ten *S. enterica* serotypes in the United States are Enteritidis, Newport, Typhimurium, Javina, I 4,[5],12:i:-, Poona, Muenchen, Heidelberg, Saint Paul, and Infantis [1]. This microorganism has adapted to survive environmental conditions such as refrigerated temperatures, harsh pH, presence of solutes, or very low moisture content in foods. The presence of *Salmonella* serotypes has been widely documented, as shown in Table 1, and this microorganism can be found in almost any kind of product.

Microbial cells usually have two different stages: planktonic and sessile cells. Planktonic cells freely exist in bulk solution; meanwhile, sessile cells are attached, usually as biofilms, presenting resistance to antimicrobials and sanitizers [2]. Biofilms represent a source of contamination, post-processing contamination, and cross-contamination in the food industry, generating several problems such as food spoilage and foodborne diseases [3]. Biofilm formation is affected by several environmental conditions, such as temperature, material of the food-contact surface, and nutrient and water availability. Information about how these conditions affect cell attachment is available for some microorganisms, but for *Salmonella* spp., there is a lack of information for understanding how the environment affects biofilm formation and elucidating how the biofilm evolves [1,4]. When cells attach to form a biofilm, these microbial layers confer some protection against certain interventions such as antimicrobials, disinfectants, UV radiation, irradiation, or cold plasma [4,5,6,7]. Yang et al. (2016) [8] showed that *S.* Enteritidis had higher resistance to inactivation with chlorine treatment (50 ppm, pH 6.8, 1 min) when the biofilm was formed in nutrient-restricted conditions at 25 °C. However, sessile cells were very sensitive to the disinfectant when the biofilm was grown at 4 °C. There are reports about the limited production of extra-polymeric substances (EPSs) that are critical for biofilm formation under specific conditions of temperature. For example, for *Salmonella* spp., it has been documented that the production of EPSs such as curli and cellulose has the highest rate when the incubation occurs between 25 °C and 28 °C [9].

The change from a planktonic to a sessile state involves modifications to gene regulation and the production of specific proteins [10]. The expression of cellulose and curli fimbriae in *Salmonella* spp., *Escherichia coli*, and potentially other *Enterobacteriaceae* is dependent on the regulator *csgD* [11,12]. The *csgD* gene is located on one of the two operons needed for curli fimbriae production, *csgDEFG.* The second operon, *csgBAC*, is induced by CsgD to express the curli fimbriae [10]. The *bcsABZC* and *bcsEFG* operons are responsible for cellulose production and are also positively regulated by CsgD through its activation of the *adrA* gene, which encodes for diguanylate cyclase. AdrA makes (3′-5′)-cyclic diguanosine monophosphate, and this messenger induces the cellulose synthase, BcsA [10,13]. The O-antigen capsule and biofilm-associated large surface protein (BapA) are also important for biofilm formation, and CsgD positively regulates the genes responsible for the expression of these proteins [10]. The EPS that are produced under the regulation of CsgD facilitate the adhesion and invasion of *Salmonella* and result in biofilm persistence in the host or on surfaces [14]. Specific genes have been shown to vary in their presence or expression among *Salmonella* serotypes [15]. These genetic differences may also impact the ability of serotypes to form biofilms.

**Table 1:** *Salmonella* strain characteristics used to study biofilm formation and removal.

*Salmonella* Strain	Source	Biosafety Level (BSL)	Related Food	References
*S*. Typhimurium 53647 (Attenuated)	ATCC * (Avirulent strain)	1		
*S*. Typhimurium 14028	ATCC	2	Peanut butter, tomatoes, cantaloupes, cucumbers, basil, alfalfa, prepackaged salads, coconut, chicken salad, ground beef	[16]
*S*. Stanley H0558	Outbreak in sprouts	2	Mushrooms, raw cashew cheese, peanuts	[16,17]
*S.* Senftenberg 8400	ATTC	2	Infant formula, peanut butter, papayas, pistachios, smoked fish, tomatoes	[16,18,19,20]
*S*. Anatum F4317	Outbreak in sprouts	2	Infant formula, basil, papaya, beef, pork, dried fish	[16,18,19,21,22]
*S.* Montevideo G4639	Outbreak in tomato	2	Raw sprouts, pistachios, tahini sesame paste, Italian meats, salmon, cheese, turkey, chicken, eggs, sausage, beef, yogurt, pork	[16,19,23]
*S*. Enteritidis PT30	Outbreak in raw almonds	2	Eggs, alfalfa, pine nuts, raw cookie dough, chicken products, peaches, bean sprouts, ground beef, cooked crab, mayonnaise, raw sausage, cake, pork, corn, potatoes, papaya, fish	[16,19,24]
*S*. Saint Paul 02-517-2	Outbreak in cantaloupe	2	Ground beef, cucumbers, alfalfa, sprouts, raw produce, dried fish, turkey meat, paprika, cantaloupes, chicken, eggs, orange juice, confectionaries, jalapeño pepper	[16,19,25]

* ATCC: American Type Culture Collection.

Although some research has been conducted on the influence of environmental factors on biofilm formation for some *Salmonella* serotypes, others, such as Anatum and Saint Paul, are rarely studied. There are still many gaps to fill in our understanding of how *Salmonella* serotypes vary in biofilm formation and how to implement the best intervention to minimize the risk of contamination. *Salmonella* spp. are microorganisms of concern in the food industry, including cold chain and warm processing conditions. Common materials in processing lines, loaders, tanks, and packers include stainless steel, polystyrene, silicone, nylon, rubber, polypropylene, and aluminum, among others. Thus, this study aimed to assess the biofilm formation of eight *Salmonella enterica* serotypes of importance in the food industry on three food-contact surfaces, stainless steel, silicone, and nylon, at two different temperatures (10 and 37 °C) and to determine if the presence or absence of certain genes is associated with biofilm behaviors.

## 2. Materials and Methods

### 2.1. Microbial Strains

Eight different *Salmonella* serotypes were used to compare the ability to form biofilms. All the microbial strains belong to the Eastern Regional Research Center Culture Collection and have different origins (Table 1). Five *Salmonella* strains are isolates from foodborne outbreaks: *S.* Anatum F4317, *S.* Enteritidis PT30, *S.* Montevideo G4639, *S.* Saint Paul 02-517-2, and *S.* Stanley H0558. Three strains are microorganisms obtained from the American Type Culture Collection (ATCC, Manassas, VA, USA): the pathogenic *S.* Senftenberg 8400 and *S.* Typhimurium 14028 and an avirulent strain, *S.* Typhimurium 53647. These strains were chosen based on their diverse origins and ability to represent the different strains in the food processing chain. All the stock cultures were kept at −80 °C in tryptic soy broth (TSB: MP Biomedicals, LLC, Solon, OH, USA) with 0.6% yeast extract (YE: Fisher bioreagents, Fair Lane, NJ, USA) and glycerol (30% *v*/*v*).

A thawed loopful from each culture was transferred to 5 mL of TSBYE and incubated overnight (24 h) at 37 °C to prepare the cells for the experiments. Afterward, 0.2 mL of the culture was transferred to 50 mL of TSBYE and incubated at 37 °C for 18 h and 160 rpm. The cells were centrifugated (15 min, 5000× *g*), washed twice (0.1% buffered peptone water: BPW, Neogen, Lansing, MI, USA), and resuspended in 9 mL of BPW; these cells were used immediately to prepare the inoculum. The viability of *Salmonella* cells in the main inoculum was tested for each experiment in xylose lysine deoxycholate (XLD, Difco^TM^, Sparks, MD, USA) agar plates incubated at 37 °C for at least 48 h.

### 2.2. Coupons

Three common food processing surfaces were tested and represented by coupons made of stainless steel (304 L), silicone, and nylon; all were made of food-grade materials. Coupons were purchased from Biosurface Technologies Corp. (Bozeman, MT, USA), and each one measured 1.27 cm (diameter) by 0.38 cm (thickness). Coupon preparation to study biofilm formation has been described in detail by Bermudez-Aguirre et al. (2025) [26]. The coupons are washed, disinfected with ethanol (70% *v*/*v*), air-dried, and sterilized in an autoclave.

### 2.3. Biofilm Formation

Biofilm formation was studied on the three food processing surfaces using the different strains of *Salmonella* inoculated in TSBYE at an initial concentration of 10^5^ cfu/mL. Polystyrene culture plates (12-well, Corning, NY, USA) were used for biofilm formation, and a coupon of each material was added to each well using sterile tweezers. The three materials were studied at the same time with the same inoculum. Then, 2 mL of inoculated TSBYE was added to each well. Afterward, the lid was added to the plate and gently shaken to ensure complete contact with the inoculum and all the coupon surfaces. For each experiment, several 12-well plates were prepared simultaneously; some plates were incubated at 10 °C and the others at 37 °C (static conditions) to study the effect of temperature on biofilm formation. Both incubator chambers (MIR-154-PA, Panasonic Healthcare Co., Ltd., Tokyo, Japan) were set up a day before the experiments. Samples were removed after 24 h for biofilm quantification.

### 2.4. Enumeration of Planktonic and Sessile Cells

After 24 h, coupons were removed from individual wells using sterile tweezers and transferred to 10 mL of sterile saline solution (0.85%) for 1 min to remove planktonic cells. This solution was kept for enumerating planktonic cells. Afterward, each coupon was transferred to 5 mL of sterile saline solution (0.85%) containing glass beads for biofilm removal. Each tube was vortexed for a minute and then processed for cell enumeration. Then, 1 mL was taken from the wash water for planktonic cells, serially diluted in BPW, and plated on Aerobic Plate Count Petrifilm^TM^ in duplicate using three dilutions. Similarly, 1 mL was taken and serially diluted for the sample containing the biofilm cells following the same procedure described above. Petrifilm was successfully validated with xylose lysine deoxycholate (XLD, Difco^TM^, Sparks, MD, USA) agar and was used in this research for logistic reasons. All the Petrifilms were incubated at 37 °C for 24–48 h and counted using a Petrifilm^TM^ Plate Reader Advanced (Neogen, Lansing, MI, USA).

### 2.5. Biofilm Index

The biofilm index (*BI*) was calculated as suggested by Nguyen et al. (2014) [2] and relates the attached cells and the planktonic cells after 24 h, as follows:

(1) $$BI=\frac{Attached\;cells\;(\log\left(cfu\;cm^{2})\right)}{Planktonic\;cells\;(\log\left(cfu\;cm^{2})\right)}$$

The *BI* was calculated for each *Salmonella* strain on each food-contact surface at each incubation temperature.

### 2.6. Sequencing

The eight *Salmonella* serotypes were each grown overnight in 10 mL of TSBYE at 37 °C. A 1.8 mL volume of each culture was placed into a 2 mL microcentrifuge tube and centrifuged at 13,000× *g* for 1 min at room temperature (21 °C). The supernatant was removed, and the tubes were centrifuged again with the same settings. The remaining supernatant was removed, and the *Salmonella* pellet was processed with a Qiagen DNeasy PowerFood Microbial Kit (Qiagen, Germantown, MD, USA) to extract DNA. A Denovix DS-11 FX + spectrophotometer (DeNovix Inc., Wilmington, DE, USA) was used to measure DNA concentration. Sequencing libraries were prepared with the Rapid Barcoding Kit (SQK-RBK114, Oxford Nanopore Technologies [ONT], Oxford, UK) using 200 ng of DNA from each sample as input. The libraries were loaded onto R10.4.1 flow cells (ONT), and long-read sequencing was performed on a GridION device (ONT). Default sequencing parameters were used in MinKNOW software (version 24.02.6, ONT), except for the following: 24 h run length, minimum quality score of 8, and minimum read length of 1 kb. The fast5 files generated during the sequencing run were base called to fastq files post-run using the high-accuracy model in MinKNOW.

### 2.7. Bioinformatics

The fastq files were uploaded to Galaxy for analysis [27]. Quality control was performed with Porechop (version 0.2.4) to remove adapters and with Fastp (version 0.23.4) using a Phred quality cutoff of 8 and minimum length of 1 kb. The data was assessed after quality control with Nanoplot (version 1.42.0). Reads were de novo assembled with Flye (version 2.9.1) in nanopore raw mode with 3 polishing iterations. The resulting assembly was polished twice with the Medaka Consensus Pipeline (version 1.7.2), and then the assembly’s quality was assessed with Quast (version 5.2.0). The assembled genomes were annotated with Prokka (version 1.14.6). Snippy (version 4.6.0) was used to identify single nucleotide polymorphisms (SNPs) in the sequencing data, and SnpEff eff (version 5.2) was used to annotate the variants. The sequencing data was also analyzed with ABRicate (version 1.0.1) using the virulence factor database to identify virulence genes.

### 2.8. Electron Microscopy

The topography of three food-contact surface materials, stainless steel, silicone, and nylon, was assessed using scanning electron microscopy. No initial preparation was required. Coupons were mounted on stubs and sputter coated with gold for 1 min (EMS 150R ES, EM Sciences, Hatfield, PA, USA). To study the biofilm formation, additional samples were prepared according to the methodology described in 2.3 using only three *Salmonella* serotypes (based on preliminary results), stainless steel coupons, and incubation at 37 °C. After 24 h, the samples were removed from the incubator and the TSBYE was replaced with 2 mL of 2% glutaraldehyde and kept at least 3 h under refrigerated conditions. Further sample preparation has been previously described by Bermudez-Aguirre et al. [26]. Samples were viewed with a FEI Quanta 200 F Scanning Electron Microscope, (Hillsboro, OR, USA) with an accelerating voltage of 10 kV in high-vacuum mode.

### 2.9. Statistical Analysis

All the experiments were conducted at least in duplicate in different weeks with a new and fresh inoculum every time. For each point, samples were taken in duplicate, and at least three dilutions were conducted and plated in duplicate. Fundamental statistical data analysis was performed using Microsoft Excel (Version 2501, Seattle, WA, USA). Analysis of variance (ANOVA—one way) was calculated using SAS (Version 9.4, Cary, NC, USA) with a confidence level of *α* 0.05 to determine any significant difference between samples. Also, a pair-wise Tukey’s test was used to find significant differences between the tested conditions using *α* 0.05.

## 3. Results and Discussion

The first part of the results will focus on the individual effects of temperature, food-contact surface, and microbial strains and discuss the biofilm index in the different conditions evaluated in the present research. The second part will focus on analyzing the results from the bioinformatics analysis of the eight *Salmonella* serotypes evaluated in this work.

### 3.1. Effect of Temperature

The effect of temperature on the biofilm formation of the eight *Salmonella* strains tested in the present research is shown in Table 2 for both temperatures. Slow biofilm formation was evident when cells were exposed to refrigerated temperatures; after 24 h, the average biofilm formation was about 5 log (cfu/cm^2^). Meanwhile, higher temperatures promoted cell growth and biofilm formation with an average of 7 log (cfu/cm^2^). According to the pair-wise Tukey’s test (a = 0.05), the effect of temperature was evident on the biofilm formation of four serotypes: *S.* Typhimurium 53647, *S.* Enteritidis, *S.* Montevideo, and *S.* Saint Paul. The mean log values for each strain were significantly different (*p* ≤ 0.05) when the incubation occurred at 10 °C and when it occurred at 37 °C. *S.* Senftenberg exhibited the most nonuniform biofilm formation in response to temperature.

The effect of temperature on biofilm formation and cell attachment has been reported for *Salmonella* spp. in some research [1,2], and the optimum temperature for bacterial attachment is 30 °C. Stepanović et al. (2003) [28] also concluded that this temperature was optimal for biofilm formation when studying 30 strains of *S. enterica* serovar Typhimurium and Enteritidis. The strongest biofilm formation was reported after 24 h at 30 °C when compared with the biofilm formed at the same time point at 22 °C and 37 °C. These authors also mentioned that the regulation pattern of the biofilm formation for *Salmonella* is a very complex process. Dhakal et al. (2019) [1] tested fifteen serotypes of *Salmonella* for biofilm formation at three different temperatures (4, 25, and 30 °C). The results showed no biofilm formation at 4 °C, but the opposite, a very dense biofilm, was observed at 30 °C. Nguyen et al. (2014) [2] reported the faster formation of a biofilm when the *Salmonella* cells were incubated at higher temperatures (28, 37, 42 °C) using a microbial growth medium (tryptic soy broth). In another study by Roy et al. (2021) [3], *S.* Kentucky was investigated for biofilm formation in different materials using a wide range of temperatures (4, 10, 25, 37, and 42 °C). The authors concluded that the optimal temperature for this serotype was between 25 and 42 °C. Obe et al. (2022) [29] studied twenty *Salmonella* strains at two different temperatures, 15 °C and 25 °C, using plastic and stainless steel. At the lower temperature, only five strains formed strong biofilms on plastic, while none formed a biofilm on stainless steel. At the higher temperature, eight of the twenty strains formed strong biofilms on plastic and three did so on stainless steel. Meanwhile, a single strain of *S.* Infantis was studied for biofilm formation from 20 °C to 40 °C, and it was found that cells transitioned faster from a planktonic to sessile state when the temperature was 30 °C and 32 °C on stainless steel [30]. It has been suggested that the optimal temperature for some *Salmonella* strains to express EPS is 28 °C, but the optimum growth temperature is 37 °C. The reduction in temperature during biofilm formation might promote the expression of other components for cell attachment [9]. The temperatures chosen in the present work represent a abuse refrigeration temperature (10 °C) and the optimal growth temperature for *Salmonella* spp. Even though 10 °C is generally thought to suppress microbial growth, it is evident, based on the present data, that *Salmonella* can quickly grow in a single medium such as TSBYE and form strong biofilms in only 24 h on food-contact surfaces that are currently used in several types of equipment in the food industry, representing a risk of cross-contamination of food products if they are exposed to these surfaces without a correct sanitizing intervention. During biofilm formation, the cells attach to the surface as layers, creating a strong biofilm. Temperature and pH influence biofilm formation because both factors regulate cell physiology, cell surface properties, microbial transcriptomic profile, nutrient availability, and the properties of the extracellular polysaccharides released by the microorganisms [3]. So, based on this information, it can be assumed that the cells undergoing biofilm formation at 10 °C exhibited a controlled and limited production of these extracellular polysaccharides, but additional compounds could be released, allowing cells to attach to the food-contact surfaces. The highest temperature (37 °C) permitted not only some production of EPSs but also the growth of *Salmonella* cells, and this is the likely reason for the difference of almost 2 logs in microbial growth between 10 and 37 °C.

### 3.2. Effect of Food-Contact Surface

Three food-contact surfaces were tested in the present research: stainless steel, silicone, and nylon. By far, stainless steel is the most commonly used material for food processing because of its unique characteristics, such as its inert nature, smooth surface, easy cleanability, and strong composition. However, all three materials can be found anywhere along the production chain. For example, silicone can be found in tubing, baking equipment, seals, packaging, and conveyor belts. Nylon is often used for crates, cutters, conveyors, and food utensils. In Table 2, the effect of the food contact surface is shown for the *Salmonella* strains at 10 °C, and stainless steel is the material with the strongest biofilm formation, followed equally by silicone and nylon. However, at 37 °C, silicone was the material with the most cells attached, followed by nylon and then stainless steel. The statistical analysis showed significant differences (*p* ≤ 0.05) between the food-contact surfaces for the following strains when cells were grown at 37 °C: *S.* Typhimurium 14028, *S.* Stanley, and *S.* Anatum. Meanwhile, the most variable serotype in its ability to form biofilms on the three different materials was *S.* Senftenberg.

**Figure 1:**
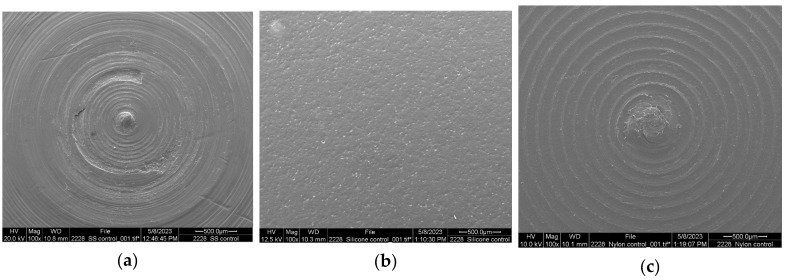
Comparison between the three food-contact surfaces for biofilm formation: (**a**) stainless steel, (**b**) silicone, (**c**) nylon. All images are with a 100× magnification.

The effect of the material of the food-contact surface has also been widely reported by several authors [1,2]. However, there are controversies about the findings and the observed differences. For example, Dhakal et al. (2019) [1] compared the biofilm formation of *Salmonella* spp. on plastic and stainless steel. Results showed that *Salmonella* grew better and formed stronger biofilms on plastic than on stainless steel. Plastic is considered a hydrophobic material, and stainless steel is a hydrophilic material. The effect of hydrophobicity on cell attachment has been reported; large numbers of cells attach to hydrophobic surfaces with little or no surface charge, moderate numbers of cells attach to hydrophobic metals with positive or neutral charge, and few cells attach to hydrophilic, negatively charged substrates [31]. In a reported study, Nguyen et al. (2014) [2] tested the biofilm formation of *Salmonella* Typhimurium on stainless steel and acrylic and assessed how temperature (28, 37, and 42 °C) and pH (6 and 7) affected the process. The results showed that *S.* Typhimurium formed stronger biofilms more quickly on stainless steel than on acrylic when the pH was 7 and the temperature was 37 °C. There is a report demonstrating a faster rate of *Salmonella* cell attachment during the early stages of biofilm formation on stainless steel but also the faster detachment of cells [29]. Roy et al. (2021) [3] discussed surface roughness as a critical factor for cell attachment. These authors mentioned that irregularities in the topography of the materials are excellent points to start microbial colonization and favor cell attachment. In a study conducted with *S.* Typhimurium using polished and unpolished stainless steel, the results showed that the microorganisms attached four times less effectively to the polished surface, and after 5 days of incubation, biofilm formation was minimal. The reason might be related to the higher concentration of metal ions. The disinfection of this electropolished surface with chlorine was 130 times more effective [32]. Images of the microstructure of the three materials tested in the present research are shown in Figure 1. Although stainless steel is considered a smooth surface, the microstructure in Figure 1a shows some irregularities that can serve as the starting point for colonization. Silicone in Figure 1b shows some porosity, and nylon in Figure 1c shows some roughness and excellent surface characteristics to form strong biofilms. These facts must be considered when choosing the proper biofilm removal and sanitization intervention in the food industry.

### 3.3. Effect of Bacterial Strain

Finally, the effect of the microbial strain is also presented in Table 2. However, environmental factors strongly affected the biofilm formation process. At 10 °C, the strongest biofilm formation on stainless steel was demonstrated by *S.* Enteritidis, while on silicone and nylon, *S.* Saint Paul formed the strongest biofilm. In contrast, the weakest biofilm-forming strain on stainless steel and silicone at 10 °C was *S.* Senftenberg, and on nylon, it was the avirulent strain *S.* Typhimurium 53647. At 37 °C, *S.* Montevideo formed the strongest biofilms regardless of the food-contact surface material. The weakest strain at this temperature was *S.* Anatum on stainless steel and *S.* Senftenberg on silicone and nylon. According to the statistical analysis and the Tukey’s test (a = 0.05), *S.* Anatum was the most different serotype in its ability to form biofilms when compared within the same material and same temperature; followed by *S.* Senftenberg, *S.* Saint Paul, and *S.* Montevideo.

Dhakal et al. (2019) [1] evaluated fifteen serotypes of *Salmonella* during biofilm formation on different materials. The results showed the highest biofilm formation on plastic materials for three ATCC strains (Typhimurium 14028, Heidelberg 8326, and Enteritidis 4931) compared to other serotypes. Meanwhile, on stainless steel, the strongest biofilm was observed for *S.* Enteritidis ATCC 4931. In another multi-serotype study, Lianou and Koustsoumanis (2012) [4] tested over 60 *Salmonella enterica* strains incubated under different pH, NaCl concentration, and temperature conditions. The effect of strain was difficult to isolate because of the other responses to environmental conditions. They concluded that about 50% of the *Salmonella* strains grew best when the pH was 5.5, NaCl was about 0.5%, and the temperature was 25 °C. Additional reports mention *S.* Agona and *S.* Montevideo as good biofilm producers and *S.* Enteritidis as one of the strongest biofilm formers with very high adhesion capabilities on glass, stainless steel, polyethylene, polystyrene, and polypropylene [9]. Obe et al. (2022) [29] tested twenty *Salmonella* serotypes related to the poultry industry and found *S.* Schwarzengrund as the strongest and *S.* Heidelberg and *S.* Newport as the weakest strains.

**Figure 2:**
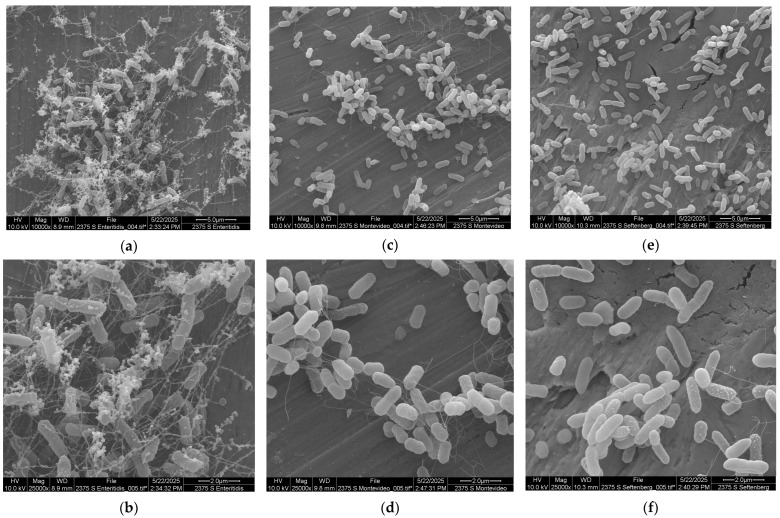
Biofilm formation of three selected *Salmonella* serotypes in stainless steel after 24 h at 37 °C: (**a**,**b**) *S. Enteritidis*, (**c**,**d**) *S. Montevideo*, (**e**,**f**) *S. Senftenberg*.

Figure 2 presents images of *Salmonella* cells forming strong and weak biofilms; all cells were allowed to start biofilm formation under the same conditions and in the same medium (TSBYE). Firstly, Figure 2a and Figure 2b present a complex network of extra-polymeric substances (EPSs) with embedded cells of *S.* Enteritidis, one of the strongest biofilm formers based on the present data and in agreement with previously reported information. Microbial cells form a mesh with the EPSs that generates a strong biofilm. The middle images (Figure 2c,d) refer to *S.* Montevideo, another strong serotype in biofilm formation, showing the presence of some appendages connecting cells. These appendages may be fimbriae or pili, flagella, or EPSs generated by the cells. Borges et al. (2018) [9] suggested that the intrinsic characteristics of each strain, such as the presence of fimbriae, flagella, membrane proteins, and other cellular appendages, are critical in biofilm formation. The last images (Figure 2e,f) show the presence of fimbriae or flagella in very few cells of *S.* Senftenberg, the weakest serotype in this study and the one that showed the most variable biofilm formation pattern. The presence of curli and cellulose has been reported in *Salmonella* biofilms as EPSs. Shatila et al. (2021) [33] presented a study with different *Salmonella* serotypes, including Enteritidis and Anatum, detecting the presence of curli and cellulose in the first strain and the absence of both compounds in the second one. These authors also mentioned the strong biofilm formation of *S.* Enteritidis and the weak attachment of *S.* Anatum on glass. The presence of curli fibers provides the biofilm with resistance to the environment during the early stages of biofilm formation. Cellulose helps to maintain the organized biofilm structure and provides protection against environmental stresses.

### 3.4. Biofilm Index (BI)

**Figure 3:**
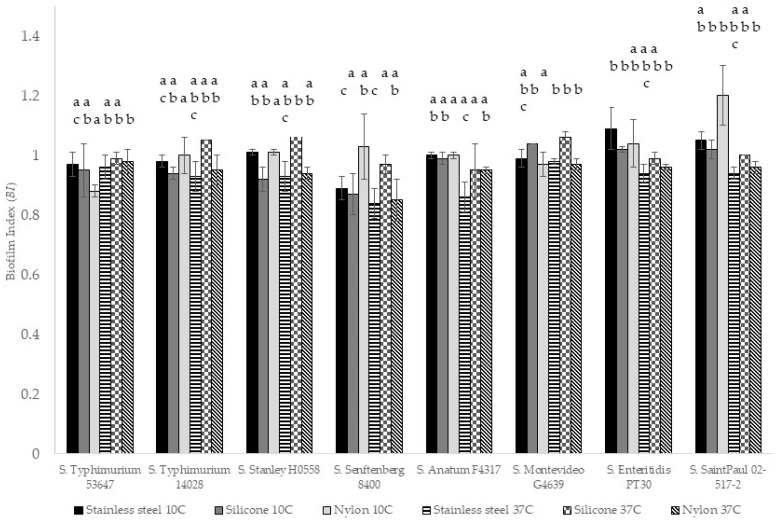
Biofilm index (*BI*) for eight *Salmonella* serotypes on three food-contact surfaces at 10 °C and 37 °C. Same letters indicate *BI* values that are statistically equal (pair-wise Tukey’s comparison a = 0.05) when compared between strains at the same temperature and on the same material.

The *BI* was calculated for each strain, food-contact surface, and temperature tested in the present research. Regarding the temperature, the *BI* was generally higher for all samples incubated at 10 °C, as shown in Figure 3. Meanwhile, regarding the food-contact surface, nylon at 10 °C showed the highest *BI*, and silicone at 37 °C had the highest value. Regarding the microbial strains, the three serovars with the highest *BI* were *S.* Montevideo, Enteritidis, and Saint Paul. The microorganism with the lowest *BI* was *S.* Senftenberg, and according to Tukey’s test (a = 0.05), this serotype was the most variable in terms of *BI*. Biofilm formation is considered an equilibrium process in the number of planktonic and attached (sessile) cells affected by environmental factors. Some free planktonic cells will attach to the biofilm, and some sessile cells will generate daughter cells that will return to the planktonic state [2]. The growth rate of planktonic cells can be affected by factors such as temperature and pH. The *BI* showed significant differences (*p* > 0.05) in stainless steel between 10 °C and 37 °C, meaning that the attached cells in both conditions were not dependent on the number of planktonic cells in the medium. According to Nguyen [2], these differences in *BI* could be caused by changes in the cell surface properties.

### 3.5. Genetic Variations

Long-read sequencing was performed on each of the eight serovars included in the study. The reads that passed quality assurance with a Phred score of at least 8 were used for further analysis, and annotated genome assemblies were generated for each *Salmonella* serovar. Genes for virulence factors were identified in each assembly using ABRicate, and the number of genes detected varied by serovar. Table 3 shows the total number of virulence genes found in each serovar and specifies whether certain genes associated with biofilm formation were present (Table 3). The sequences for each serovar were also compared to the annotated genome assembly of *S.* Stanely to determine if there were single nucleotide polymorphisms (SNPs) present that may account for biofilm capability (Table 4).

*S.* Montevideo was one of the strongest biofilm formers in this study, regardless of the food-contact surface material, but it did not have the *lpf* genes that were found in the other serovars. The chromosomal *lpfABCDE* operon contains genes that encode the subunits of the long polar fimbriae that adhere to M cells in Peyer’s patches [14] and can contribute to biofilm formation [34]. Members of *Salmonella enterica* subspecies I clade 4, of which *S.* Montevideo is a member, do not possess *lpf* genes [35]. However, *Salmonella* serovars have an average of 12 fimbrial gene clusters; therefore, the absence of a particular fimbria could be complemented by others [36]. Fimbrial *fae* genes are infrequently found in *Salmonella* serovars, but *faeC*, *faeD*, and *faeE* were identified in *S.* Montevideo, as well as *S.* Stanley and *S.* Saint Paul [36]. Other studies have suggested that possessing *fae* genes may contribute to host range expansion or improved colonization in specific hosts [36]. In this study, the Fae fimbriae may have enhanced biofilm formation on the food-contact surfaces.

In contrast to *S.* Montevideo, *S.* Senftenberg was one of the weakest biofilm formers, but it did possess the *lpf* operon. However, there were five noncoding variations and one synonymous variation identified in *lpfA*. The synonymous variation should not have altered amino acids in LpfA or changes in its function. The SNPs in the noncoding regions may affect regulation of LpfA and potentially reduce adherence and biofilm formation. Additional research is needed to determine if the SNPs in *lpfA* account for weaker biofilm formation in *S.* Senftenberg.

Genetic variations identified in the curli genes may be the most consequential to determining whether *Salmonella* serovars form strong biofilms. Nearly all *Salmonella* produce curli, thin, aggregative, proteinaceous fibers that mediate adherence, invasion, cell aggregation, and biofilm formation [37]. The curli genes are organized in two operons, *csgDEFG* and *csgBAC* [13]. Three serovars had SNPs in at least one of the curli genes. *S.* Senftenberg had one noncoding variant in *csgF.* Alterations in the regulation of *csgF* may have reduced curli expression and resulted in a weaker biofilm in this serovar. Two of the strongest biofilm formers, *S.* Saint Paul and *S.* Montevideo, also had SNPs in *csg* genes. In *csgB*, S. Saint Paul had one missense variant and *S.* Montevideo had two missense variants identified. *S.* Montevideo also had two missense and one synonymous variant in *csgC.* The missense variants in the *csg* genes may have altered the proteins in a manner that resulted in stronger biofilms. Further research to determine how the SNPs in each of these serovars impact curli formation could provide insight into why different serovars vary in biofilm formation.

## 4. Conclusions

Biofilm formation is a very complex process that is highly affected by environmental conditions, food-contact surface material, and *Salmonella* serovar. In the present research, *Salmonella* formed stronger biofilms at 37 °C after 24 h. The food-contact surface material also affected biofilm formation, with stronger biofilms forming on stainless steel at low temperatures and silicone at higher temperatures. *S.* Enteritidis, *S.* Saint Paul, and *S.* Montevideo were the strongest serotypes forming biofilms, while *S.* Senftenberg was generally the weakest. Single nucleotide polymorphisms identified in *csg* genes of *S.* Saint Paul, *S.* Montevideo, and *S.* Senftenberg may contribute to the differences observed in biofilm formation strength in these serovars.

Further research is recommended to study the biofilm formation of *Salmonella* spp. in more *real-case-scenario* conditions. In the study of *Salmonella* spp. in combination with common microorganisms found in the food processing environment such as *Pseudomonas* spp., the use of complex media (i.e., food debris) to start the biofilm formation or of intermittent incubation temperatures might bring additional insights. The information from the present research can help to develop adequate interventions for very diverse food processing environments and reduce the incidence of this foodborne pathogen in post- and cross-contamination incidents.

## Figures and Tables

**Table 2:** Results of biofilm formation of *Salmonella* strains at two different temperatures (10 °C and 37 °C) in three different materials.

10 °C	Log N	Log N	Log N
	Stainless Steel	Silicone	Nylon
*S.* Typhimurium 53647	5.16 ^abA^ (±0.28)	5.08 ^abA^ (±0.63)	4.53 ^aA^ (±0.13)
*S*. Typhimurium 14028	5.06 ^abC^ (±0.14)	5.00 ^abC^ (±0.22)	4.80 ^aC^ (±0.47)
*S.* Stanley H0558	5.44 ^abB^ (±0.37)	4.89 ^abB^ (±0.33)	5.29 ^abB^ (±0.16)
*S.* Senftenberg 8400	4.77 ^aCD^ (±0.42)	4.26 ^aC^ (±0.81)	4.83 ^abACD^ (±0.42)
*S.* Anatum F4317	5.36 ^abAC^ (±0.18)	5.23 ^abAC^ (±0.13)	4.93 ^abA^ (±0.29)
*S.* Montevideo G4639	5.37 ^abA^ (±0.16)	5.43 ^bA^ (±0.12)	5.15 ^abA^ (±0.04)
*S*. Enteritidis PT30	5.53 ^bA^ (±0.11)	5.27 ^abA^ (±0.30)	5.27 ^abA^ (±0.30)
*S.* SaintPaul 02-517-2	5.49 ^bA^ (±0.01)	5.47 ^bA^ (±0.15)	5.56 ^bA^ (±0.13)
**37 °C**			
*S.* Typhimurium 53647	7.15 ^abB^ (±0.37)	7.30 ^abB^ (±0.17)	7.28 ^bB^ (±0.30)
*S*. Typhimurium 14028	6.65 ^abcA^ (±0.44)	7.62 ^bB^ (±0.04)	7.11 ^abAB^ (±0.23)
*S.* Stanley H0558	6.90 ^abcA^ (±0.19)	7.72 ^b^ (±0.05)	7.01 ^abA^ (±0.02)
*S*. Senftenberg 8400	6.20 ^acABD^ (±0.58)	6.93 ^aB^ (±0.29)	6.31 ^aAB^ (±0.68)
*S*. Anatum F4317	6.03 ^cC^ (±0.43)	6.96 ^aB^ (±0.52)	6.91 ^abB^ (±0.13)
*S.* Montevideo G4639	7.37 ^bB^ (±0.28)	7.78 ^bB^ (±0.07)	7.29 ^bB^ (±0.01)
*S*. Enteritidis PT30	7.19 ^bB^ (±0.08)	7.33 ^abB^ (±0.00)	7.27 ^bB^ (±0.01)
*S.* SaintPaul 02-517-2	7.06 ^abB^ (±0.03)	7.43 ^abB^ (±0.18)	7.23 ^bB^ (±0.25)

Values represent the average of at least two experiments ± standard deviation. ^a–c^ Equal lowercase letters indicate statistically equal means (Tukey’s test a = 0.05) between strains when compared with the same material and same temperature. ^A–D^ Equal uppercase letters indicate statistically equal means (Tukey’s test a = 0.05) between materials when compared with the same bacterial strain and both temperatures.

**Table 3:** Number of virulence factors identified in each *Salmonella* serovar.

Serovar	Number	faeC	faeD	faeE	lpfA	lpfB	lpfC	lpfE
*S.* Typhimurium 53647 (Attenuated)	117	−	−	−	+	+	+	+
*S.* Typhimurium 14028	118	−	−	−	+	+	+	+
*S.* Stanley H0558	105	+	+	+	+	+	+	+
*S.* Senftenberg 8400	101	−	−	−	+	+	+	+
*S.* Anatum F4317	111	−	−	−	+	+	+	+
*S.* Montevideo G4639	99	+	+	+	−	−	−	−
*S.* Enteritidis PT30	112	−	−	−	+	+	+	+
*S.* Saint Paul 02-517-2	109	+	+	+	+	+	+	+

**Table 4:** The type and number of single nucleotide polymorphisms identified in genes associated with biofilm formation in the *Salmonella* serovars in this study.

Strain	Gene	Single Nucleotide Polymorphism Type
*S.* Typhimurium 53647 (Attenuated)		
*S.* Typhimurium 14028		
*S.* Stanley H0558		
*S.* Senftenberg 8400	csgF lpfA	1 noncoding 5 noncoding, 1 synonymous
*S.* Anatum F4317		
*S.* Montevideo G4639	csgB csgC	2 missense 2 missense, 1 synonymous
*S.* Enteritidis PT30		
*S.* Saint Paul 02-517-2	csgB	1 missense

## Data Availability

The original contributions presented in this study are included in the article. Further inquiries can be directed to the corresponding author.

## Statement

The mention of trade names or commercial products in this article is solely to provide specific information. It does not imply a recommendation or endorsement by the U.S. Department of Agriculture. The USDA is an equal opportunity provider and employer.

## Abbreviations

The following abbreviations are used in this manuscript:

ATCC	American Type Culture Collection
BI	Biofilm index
BPW	Buffered peptone water
cfu	Colony-forming units
DNA	Deoxyribonucleic acid
g	Gravity
mL	Milliliter
µL	Microliter
ng	Nanogram
ONT	Oxford Nanopore Technologies
rpm	Revolutions per minute
SNP	Single nucleotide polymorphism
TSB	Tryptic soy broth
TSBYE	Tryptic soy broth with 0.6% yeast extract
XLD	Xylose lysine deoxycholate
YE	Yeast extract
